# Tunability of Amorphous MoS_2_ Thin Film Properties Through Pulsed KrF Laser Deposition Rate

**DOI:** 10.1021/acsami.6c00013

**Published:** 2026-03-23

**Authors:** Milos Krbal, Jan Prikryl, Vit Prokop, Jhonatan Rodriguez Pereira, Stanislav Slang, Jan Mistrik, Igor Pis

**Affiliations:** † Center of Materials and Nanotechnologies (CEMNAT), Faculty of Chemical Technology, 48252 University of Pardubice, Legions Square 565, 530 02 Pardubice, Czech Republic; ‡ Institute of Applied Physics and Mathematics, Faculty of Chemical Technology, University of Pardubice, Studentska 95, Pardubice 53210, Czech Republic; § Institute of Electrical Engineering, 87171 Slovak Academy of Sciences, Dubravska cesta 9, 841 04 Bratislava, Slovakia

**Keywords:** amorphous thin films, PLD, MoS_2+*x*_, MoS_2_, optical properties, XPS, photothermal conversion

## Abstract

In this study, we demonstrate how the physical properties of amorphous thin MoS_*x*_ films can be controlled through pulsed laser deposition conditions. We show that the sulfur content in thin MoS_*x*_ films can be specifically tuned from MoS_4_ to MoS_2_ by the laser fluence in the range of 1.3 to 20.4 J/cm^2^ using a repetition rate of 1 Hz. Focusing on stoichiometric amorphous thin MoS_2_ films, we further found that while the stoichiometry of MoS_2_ is maintained at different repetition rates (1, 3, 5, and 10 Hz) of a pulsed KrF laser, X-ray photoelectron spectroscopy reveals a significant increase in the ratio of 1T′/2H structural units as the laser repetition rate increases. This structural shift is attributed to the increased adatom mobility and cumulative surface heating occurring at higher repetition rates, which favor the stabilization of the metastable 1T′ phase while simultaneously promoting the desorption of excess polysulfides and surface molybdenum oxide impurities. Consequently, the optical parameters (refractive index and extinction coefficient) of amorphous thin MoS_2_ films show a pronounced increase with laser frequencies ranging from 1 to 10 Hz. Furthermore, we show that higher photothermal conversion is systematically associated with an increased extinction coefficient. These findings are expected to advance both fundamental research and application-driven studies, including photothermal cancer therapy, sterilization, and disinfection.

## Graphical abstract

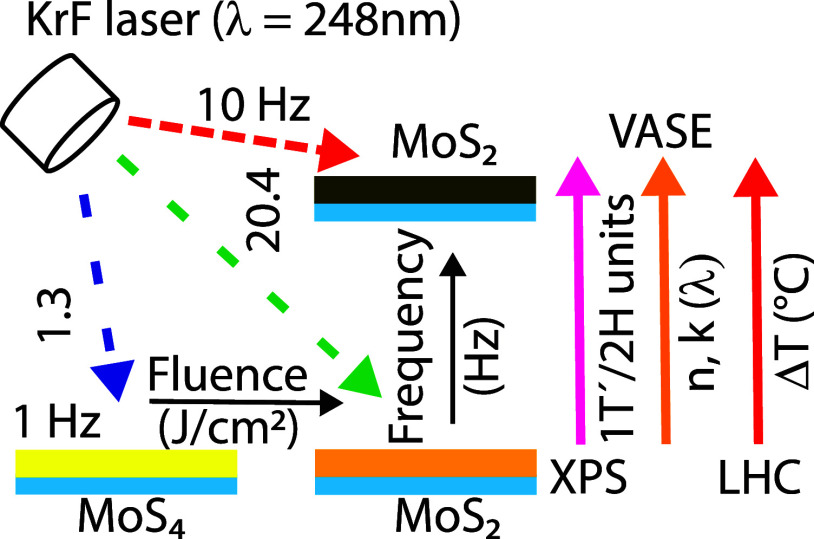

## Introduction

Transition metal dichalcogenides (TMDCs) are a versatile class of materials,^1,2^ available across a broad spectrum of morphologies, including bulk crystals, thin films, two-dimensional (2D) graphene-like monolayers,^1^ nanoparticles,^3^ nanotubes,^4^ nanospheres,^4,5^ and nanowires,^6^ existing in both crystalline and amorphous states.^3,7–9^ The morphology and polymorphic phase of TMDCs fundamentally dictate the resulting material properties, positioning TMDCs as essential components in numerous advanced applications, spanning fields such as intrinsic solid lubrication,^10,11^ advanced optoelectronics,^12^ highly sensitive biosensing,^13,14^ efficient photocurrent generation,^15^ photocatalytic degradation of organic pollutants,^16^ electrocatalytic hydrogen evolution,^17^ and cutting-edge energy storage technologies.^18^

While TMDCs are widely studied in the crystalline 2D or a bulk phase, the amorphous TMDC phases offer many interesting properties, which differ significantly from the crystalline phases. This can be demonstrated on MoS_2_, which is a typical representative of this class materials. For example, amorphous stoichiometric MoS_2_ contains a large amount of homopolar Mo–Mo bonds, which dramatically change its optical properties including, a band gap which is ≈0.2 eV compared to ≈1.1 eV for bulk crystalline MoS_2_ in the 2H polymorph.^9^ Due to the formation of the close-packed Mo clusters that exceed the conductive percolation threshold value, the amorphous MoS_2_ phase has 3 orders of magnitude higher conductivity in contrast to the crystalline 2H MoS_2_ phase.^19^ As shown, a combination of this enormous changes in properties with high crystallization temperature, stoichiometric MoS_2_ can be considered strong candidate for applications in data storage, similarly to the CrGeTe alloy.^20^ It is worth mentioning that amorphous TMDC nanoparticles, prepared by various processes, can be a cheap and effective successor to crystalline TMDC in applications related to electrocatalytic hydrogen evolution.^21–23^

The use of physical vapor deposition, such as the pulsed laser deposition (PLD) technique, offers important advantages, including thickness control using laser repetition rates, a wide range of laser energy output, and deposition under different gases and their pressures. In addition, the surface activation of the substrates is improved due to the often activated ablated fragments impinging on the substrates, which enhances the surface chemistry. As a result, compact high-quality thin films through high reaction rates can be obtained.^24^ The chemical composition, stoichiometry and morphology of the prepared thin films depend on several well-adjustable variables, which are crucial for obtaining layers with the desired properties.^25–28^ Up to date, the stoichiometric deposition of amorphous MoS_2_ thin films from the crystalline target of the same composition remains challenging. Recent studies have clearly shown that amorphous Mo–S thin films prepared using the PLD technique contain a significant excess of sulfur.^28^ On the other hand, it has been reported that amorphous sulfur-depleted thin MoS_*x*_ films (1 < *x* < 2) can be produced from a stoichiometric MoS_2_ target at room temperature using a Nd:YAG pulsed laser operating at the wavelength of 1064 nm with a pulse duration of 5 ns.^29^ A study of PLD deposition conditions required to obtain composition and quality of the amorphous TMDC film is therefore highly desirable to ensure their proper use.

In this work, we present the preparation of amorphous MoS_*x*_ thin films on the route from sulfur-rich Mo–S represented by MoS_4_ to stoichiometric MoS_2_ using pulsed laser deposition with a KrF pulsed laser as a function of the laser fluence under constant pulse frequency of 1 Hz at room temperature. We further demonstrate that the ratio of 1T′/2H structural units in the stoichiometric amorphous thin MoS_2_ films can be accurately controlled varying the laser pulse frequency from 1 to 10 Hz at constant fluence, as well as its impact on optical properties of the deposited amorphous MoS_2_ thin films, all from the same MoS_2_ target.

## Methodology

Amorphous MoS_*x*_ (*x* = 2–4) thin films were deposited at room temperature by pulsed laser deposition in an off-axis geometry using a KrF laser with a wavelength of 248 nm and a pulse duration of 30 ns. A distance between a MoS_2_ target and a substrate holder was ≈4 cm. For deposition of MoS_*x*_ thin films, the laser frequency was set to 1 Hz while the laser fluence was varied from 1.3 J/cm^2^ to 20.4 J/cm^2^. The laser frequency dependence on the MoS_2_ structure was employed in the range from 1 to 10 Hz at the constant laser fluence of 20.4 J/cm^2^. All depositions were performed under a residual pressure 2.8 × 10^–4^ Pa. A silicon wafer with (100) crystallographic orientation and fused silica were used as substrates. The thickness of the as-deposited films was approximately 50 nm, which corresponds to about 1500 pulses. Prior to opening the chamber, the deposited samples remained under vacuum for an additional 2 h to cool down and release tension.

The amorphous character of the MoS_*x*_ thin films was confirmed by X-ray diffraction using a diffractometer (Empyrean Malvern Panalytical) with Cu Kα (λ = 1.5406 Å) X-ray source in a grazing incidence geometry at a glancing angle of 0.5°.

The surface morphology was investigated by scanning electron microscopy (SEM) using a LYRA3 (Tescan) with an accelerating voltage of 10 kV. Quantitative elemental analysis was performed using energy dispersive X-ray spectroscopy (EDX), carried out on the same microscope equipped with an AZtec X-Max 20 analyzer (Oxford Instruments) at a lower accelerating voltage of 5 kV. The overall composition was determined by averaging measurements from five distinct spots.

The surface chemical composition was analyzed using X-ray photoelectron spectroscopy (XPS, ESCA2SR, Scienta-Omicron) with a monochromatized Al Kα X-ray source. Binding energies were calibrated to the adventitious carbon C 1s peak at 284.8 eV, and the total instrumental energy resolution was 0.5 eV. The S 2p and S 2s core-level spectra were fitted with Voigt functions, while Gaussian–Lorentzian product pseudo-Voigt functions provided better results for the Mo 3d spectra. A Shirley-type background correction was applied to both core-level spectra. Spin–orbit doublet separations (Mo 3d_5/2_–Mo 3d_3/2_ and S 2p_3/2_–S 2p_1/2_) and intensity ratios were calibrated using a reference 2H-MoS_2_ powder sample. The Mo 3d_3/2_ peaks appeared slightly broader than the Mo 3d_5/2_ peaks due to the Coster–Kronig effect. Relative atomic concentrations were determined from the peak areas normalized to the corresponding relative sensitivity factors obtained from the reference MoS_2_ powder sample.

Ellipsometric spectra were recorded by VASE ellipsometer (Woollam Co. Ltd.) in spectral range 0.7–6.5 eV. Angle of incidence from 50° to 80° was spanned equidistantly by 10°. Obtained spectra were treated simultaneously with optical reflectivity measured by the same instrument. Sample model consisted of c-Si semi-infinitive substrate covered by SiO_2_ native oxide and an amorphous MoS_2_. The model dielectric function for MoS_*x*_ consists of a sum of Lorentz oscillators and a pole that accounts for the contribution (to the real part of the electric permittivity) of other electronic transitions far in the UV, outside the measured spectral range. The number of Lorentz oscillators used is 1, 3, 3, and 2 for MoS_*x*_ deposited with frequency rates of 1, 3, 5, and 10 Hz, respectively. Surface roughness was also considered by means of Bruggeman effective medium with a mixture of 50% voids and 50% MoS_2_.

Light-heat conversion in selected MoS_2_ films was evaluated using a supercontinuum laser to simulate the near-infrared (NIR) therapeutic window. The films were exposed to a collimated, 4 mm diameter, polychromatic beam (1000–2200 nm) with a total power of 340 mW at a 45° angle of incidence. A long-pass filter was used to eliminate wavelengths below 1000 nm. The resulting temperature increase was monitored with an FLIR i7 infrared camera, with the film emissivity set to 0.6. NIR-transparent fused silica substrates were used to prevent substrate light absorption and heat generation.

## Results and Discussion

As mentioned in the Introduction, the deposition of the stoichiometric amorphous MoS_2_ from a stoichiometric MoS_2_ target using PLD is a nontrivial process. During PLD, the laser pulse interacts with the target material, and lighter, more volatile elements like sulfur are more efficiently ejected into the vapor plume, leading to a significant sulfur excess in the deposited MoS_*x*_ thin films.^28^

To determine the optimum laser fluence for the deposition of stoichiometric amorphous MoS_2_, the laser frequency was initially fixed at 1 Hz. The compositional dependence of amorphous MoS_*x*_ thin films on the laser fluence is shown in Figure 1. As shown, the sulfur content in the deposited films gradually (exponentially) decreases with increasing laser fluence starting from a sulfur-rich MoS_4_ composition at 1.3 J/cm^2^ and reaching the stoichiometric MoS_2_ composition at 17.5 J/cm^2^. An additional increase in laser fluence to 20.4 J/cm^2^ does not induce any further changes in the stoichiometry of the amorphous MoS_2_ thin films. The molybdenum and sulfur concentrations in the deposited amorphous MoS_*x*_ thin films, along with the corresponding laser output energies, fluences, and spot areas are summarized in Table 1.

**Figure 1:**
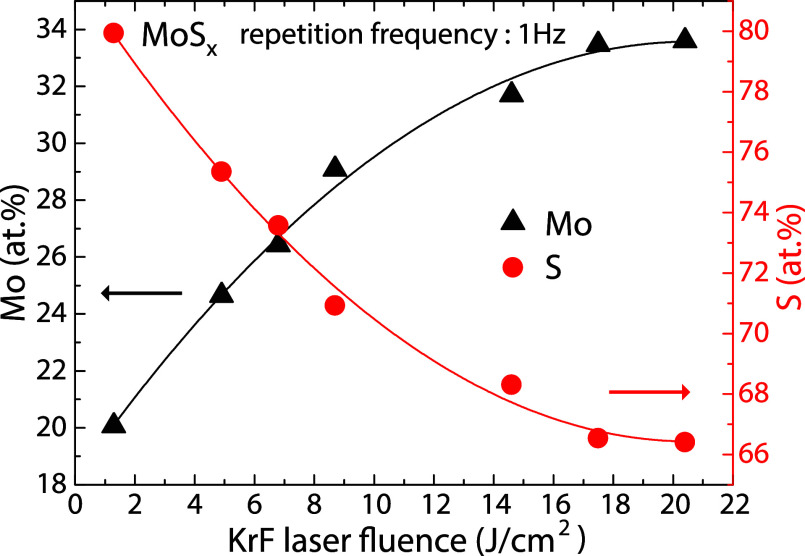
(Color online) Compositional dependence of the as-deposited thin Mo–S films on the KrF pulsed laser fluence varied from 1.3 J/cm^2^ to 20.4 J/cm^2^ at a constant laser frequency of 1 Hz.

**Table 1:** KrF Laser Output Energy (*E*), Laser Fluence (*I*), Laser Spot Area (*S*), and the Molybdenum and Sulfur Concentrations in Deposited Amorphous MoS_*x*_ Thin Films

*E* (mJ)	*I* (J/cm^2^)	*S* (cm^2^)	Mo (% at.)	S (% at.)
350	20.4	0.245 × 0.07	33.5	66.5
300	17.5	0.245 × 0.07	33.6	66.4
250	14.6	0.245 × 0.07	31.7	68.3
150	8.7	0.245 × 0.07	29.1	70.9
340	6.8	0.37 × 0.135	26.4	73.6
200	4.9	0.34 × 0.12	24.6	75.4
210	1.3	0.63 × 0.25	20.1	79.9

Figure 2 provides evidence of the amorphous structure of the as-deposited MoS_*x*_ thin films. All X-ray diffraction (XRD) patterns, specifically measured at a glancing angle of 0.5°, show the complete absence of any sharp, distinct Bragg peaks. Instead, the presence of a broad, featureless background confirms that the material lacks long-range crystalline order.

**Figure 2:**
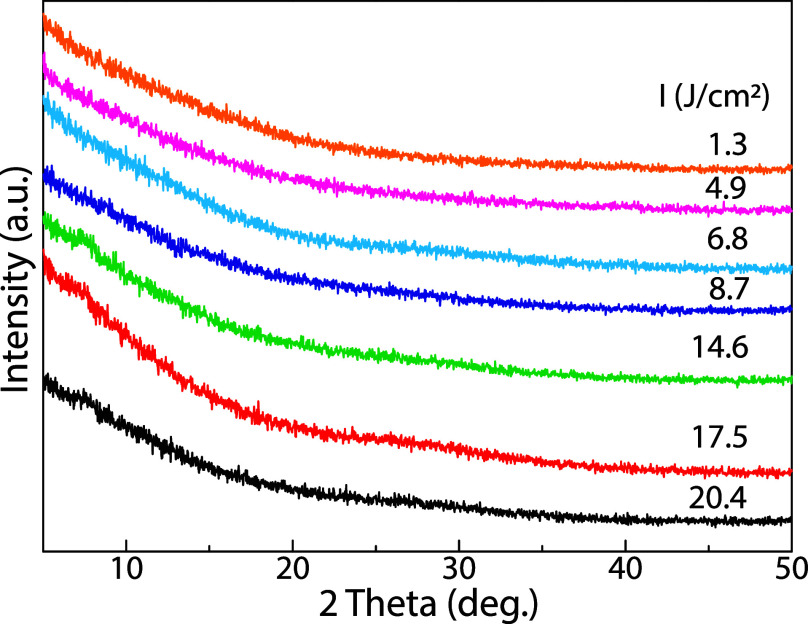
(Color online) XRD patterns of the as-deposited MoS_*x*_ thin films under the varied fluence from 1.3 J/cm^2^ to 20.4 J/cm^2^ at a constant laser frequency of 1 Hz.

The surface chemical composition of the as-deposited amorphous MoS_*x*_ films was analyzed using XPS. The Mo 3d, S 2s, and S 2p spectra, along with the applied peak deconvolution, are shown in Figure 3. The Mo 3d_5/2_ peak (Figure 3A) was decomposed into two principal components at binding energies of 229.4 ± 0.05 eV and 228.9 ± 0.15 eV, attributed to structural units representing 2H-MoS_2_ and 1T′-like MoS_2_ phases, respectively.^19,30,31^ Weaker Mo 3d components, with Mo 3d_5/2_ peaks centered at 232.3 ± 0.2 eV and 230.2 ± 0.2 eV, are attributed to surface molybdenum oxides and suboxides.^32^ The component at 230.2 eV may also arise from molybdenum oxo-sulfides^33,34^ and MoS_2_ satellites, which result from final-state screening effects.^30,35^ The components at lower binding energies originate from the S 2s core level, which we deconvoluted into a polysulfide peak at 227.36 ± 0.25 eV and a peak at 226.3 eV, corresponding to the superposition of the two MoS_2_ phases.^36^

**Figure 3:**
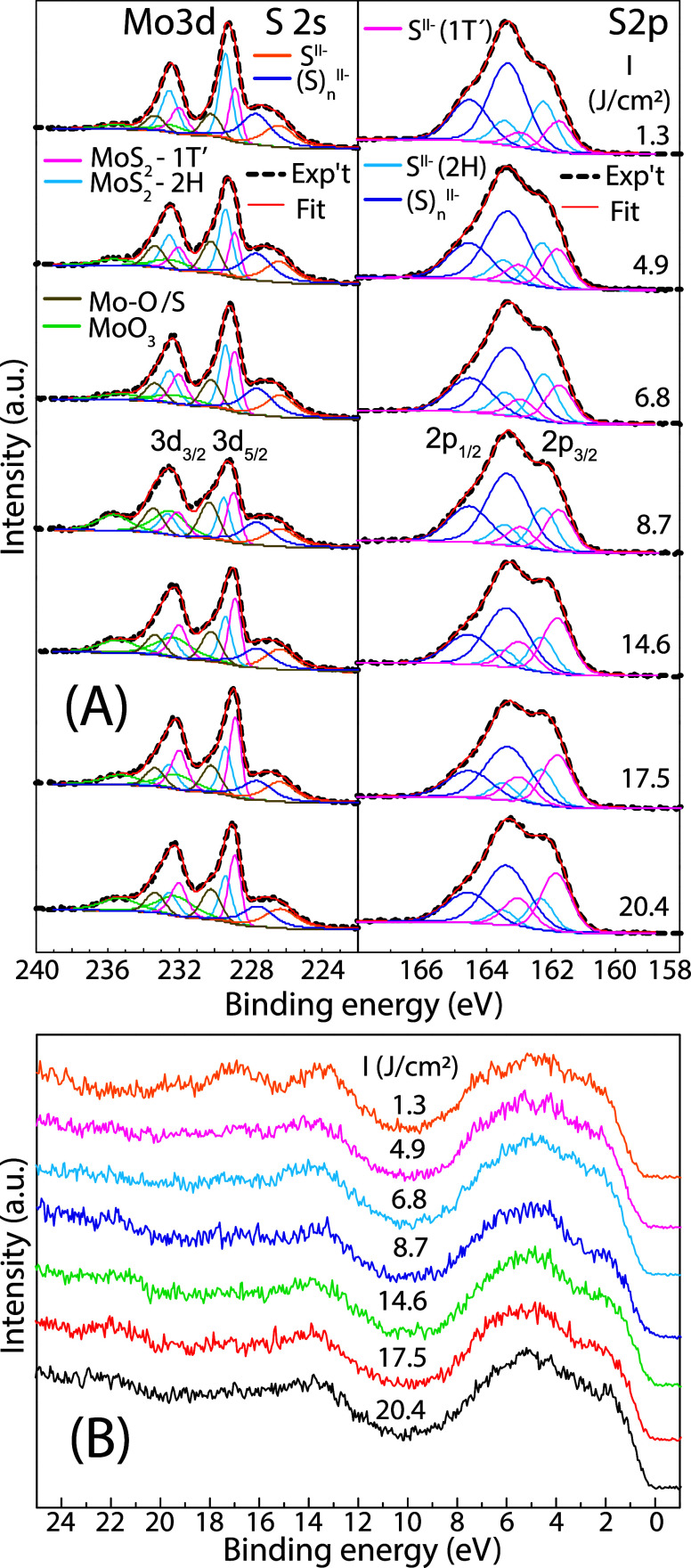
(Color online) (A) Mo 3d and S 2p XPS spectra and (B) valence bands of the as-deposited MoS_*x*_ thin films under the varied fluence from 1.3 J/cm^2^ to 20.4 J/cm^2^ at constant laser frequency of 1 Hz.

The S 2p spectral were fitted using three S 2p_3/2_-S 2p_1/2_ spin–orbit doublets, attributed to 2H-MoS_2_, 1T′-like MoS_2_,^36^ and polysulfide chains,^37^ with S 2p_3/2_ peak components at binding energies of 162.3 ± 0.1 eV, 161.8 ± 0.1 eV, and 163.3 ± 0.2 eV, respectively. The percentage contributions of the Mo 3d and S 2p components in amorphous MoS_4_, corresponding to the different chemical states, are summarized in Table 2.

**Table 2:** Percentage Contributions (at. %) of Individual Components to the XPS Signal of the As-Deposited Amorphous MoS_*x*_ Deposited over a Range of Laser Fluences from 1.3 to 20.4 (J/cm^2^)­^a^

*I* (J/cm^2^)	Mo–S (1T′)	Mo–S (2H)	Mo–O/S	Mo–O MoO_3_	S(-II) (1T′)	S(-II) (2H)	S_*n*_(-II)
20.4	10.5	6.9	5.7	6.7	22.1	13.2	34.8
17.5	9.3	6.6	6.3	8.9	23.1	10.8	34.9
14.6	8.9	6.7	6.2	8.9	22.5	12.4	34.5
8.7	7.0	6.1	7.5	9.1	15.3	15.4	39.8
6.8	7.0	8.4	5.1	4.2	15.0	17.7	42.7
4.9	5.1	9.2	5.9	3.8	15.5	16.7	43.8
1.3	5.7	10.4	3.9	2.9	12.4	18.9	45.7

a The relative contributions were calculated from integrated intensities after normalization to the corresponding Mo 3d and S 2p sensitivity factors.

Differences between amorphous MoS_*x*_ can also be identified by comparing their valence band (VB) spectra, shown in Figure 3B. In the binding energy range of 0–10 eV,^38,39^ the spectra are dominated by the hybridization of Mo 4d and S 3p states. The VB spectra do not differ significantly from one another, but the VB maximum, determined by extrapolating the leading edge of the VB at the lowest binding energies, gradually shifts from 0.40 ± 0.05 eV to 0.25 ± 0.05 eV with increasing laser fluence.

The higher concentration of polysulfides observed in the surface region of PLD-deposited amorphous MoS_2_ films, compared to previously reported magnetron-sputtered amorphous MoS_2_,^9^ motivated us to investigate the influence of deposition parameters on MoS_2_ film composition. Since an increase in laser fluence beyond 17.5 J/cm^2^ no longer alters the film composition, the laser pulse repetition rate was examined as an additional parameter potentially affecting the compactness and surface composition of the deposited films. The repetition rate was gradually increased from 1 Hz to 3, 5, and 10 Hz. At 10 Hz, the laser repetition rate is low enough to ensure that the plasma plume has sufficient time to fully expand and dissipate before the subsequent pulse. This minimizes plume–plume interactions,^40^ which are known to compromise film uniformity and induce defects. On the other hand, high repetition rate can trigger the formation of droplets on the surface of PLD thin films, primarily due to thermal accumulation in the target.^41^ This accumulation causes nonequilibrium ablation, specifically promoting mechanisms like subsurface explosive boiling and the mechanical splashing of molten material caused by the interaction with consecutive high-energy laser pulses. These macroscopic particles are propelled from the target alongside the atomic species in the plasma plume, subsequently degrading the surface quality.

The surface morphology and the elemental composition of the thin film were investigated using SEM coupled with EDX. Elemental analysis indicated that all thin films deposited across the tested range of repetition rates were stoichiometric MoS_2_, falling within the standard deviation of the method.

The surface morphology screening revealed a negligible amount of droplets on the surface of the thin films deposited at laser repetition rates of 1, 3, and 5 Hz. In contrast, deposition at 10 Hz generated a significant coverage of the film by particulates with different sizes, which can be directly associated with hydrodynamic sputtering, a phenomenon characteristic of nonequilibrium ablation.^24,41,42^ The droplet formation can potentially be mitigated by fast rotation of the target and/or by laser beam rastering over a large target area, which prevents excessive and localized overheating.

Figure 5 confirms the amorphous nature of the as-deposited MoS_2_ thin films, as measured by XRD using a glancing angle of 0.5°. A closer examination reveals that the XRD pattern of MoS_2_ deposited at 1 Hz appears featureless, while broad bands, which are typical of an amorphous phase with short- and medium-range order, become evident at higher laser repetition rates. Additionally, a faint diffraction peak around 40° emerges in the XRD patterns of films deposited at 5 and 10 Hz, attributed to Mo(110).^43^ However, its intensity is comparable to the noise level. We suggest that these differences in the XRD patterns are related to the local overheating of the topmost film by impacting fragments from the plume. At higher laser repetition rates, the substrate experiences more frequent energetic pulses from the plasma plume, resulting in cumulative substrate heating and elevated transient surface temperatures. This localized thermal spike enhances adatom mobility, allowing sputtered species to diffuse and rearrange into a denser packing configuration. This effect increases the film density and is capable of inducing subtle structural ordering even within a predominantly amorphous matrix. Moreover, it may simultaneously contribute to a reduction in the sulfur content at the surface of the film.

**Figure 4:**
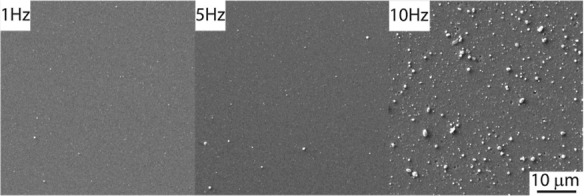
(Color online) SEM images of the as-deposited MoS_2_ thin films deposited at 1, 5, and 10 Hz.

**Figure 5:**
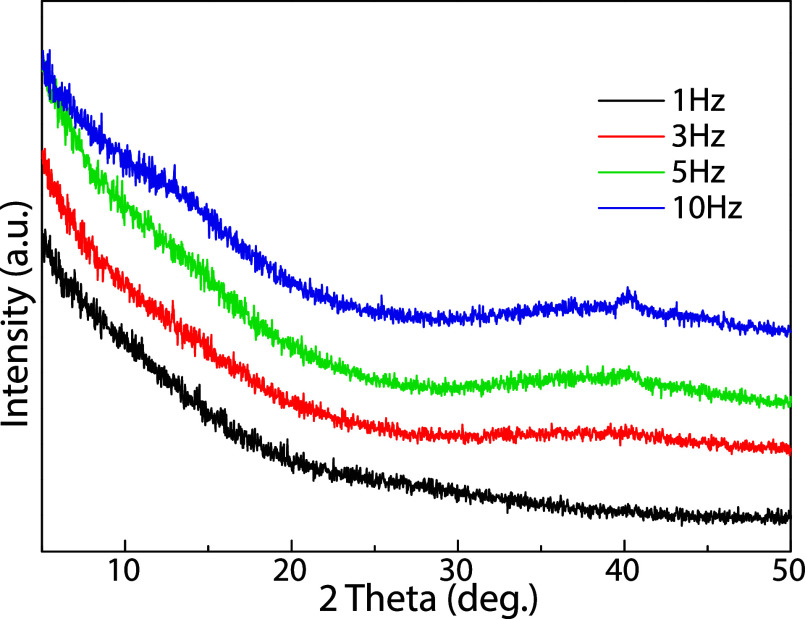
(Color online) XRD patterns of the as-deposited MoS_2_ thin films deposited at 1, 3, 5, and 10 Hz.

To further assess the influence of the repetition rate on the surface composition, XPS was used to analyze the as-deposited MoS_2_ layers. Figure 6A shows Mo 3d and S 2p spectra of MoS_2_ amorphous films deposited at 1, 3, 5, and 10 Hz. Following the spectral peak analysis described above, the percentage contributions of the chemical states for all MoS_2_ films are summarized in Table 3. The Mo 3d and S 2p peak analyses indicate that the relative amount of the 1T′-like MoS_2_ phase increases significantly as the laser repetition rate in pulsed laser deposition is raised from 1 to 3 and 5 Hz, as shown in Table 4. A repetition rate of 10 Hz further increased this ratio, though the difference between 5 and 10 Hz was smaller compared to the changes observed at lower rates. In addition, increasing the repetition rate reduces undesirable phases, specifically molybdenum oxide surface impurities and polysulfides that do not form Mo–S bonds.

**Figure 6:**
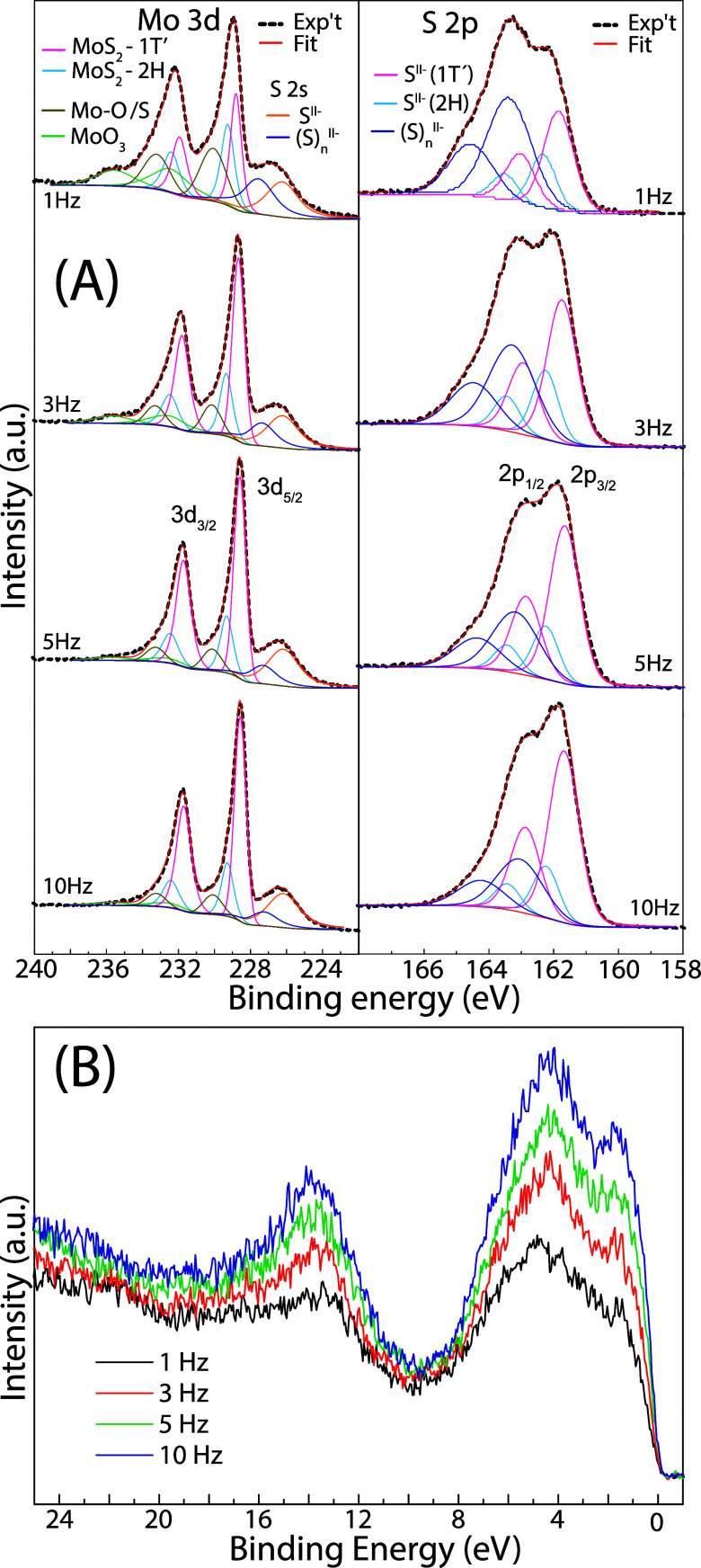
(A) Mo 3d and S 2p XPS spectra and (B) valence bands of the MoS_2_ thin films deposited at 1, 3, 5, and 10 Hz.

**Table 3:** Percentage Contribution (at. %) of Individual Components to the XPS Signal of the As-Deposited Amorphous MoS_2_ Deposited at Repetition Rates 1, 3, 5, and 10 Hz^a^

frequency (Hz)	Mo–S (1T′)	Mo–S (2H)	Mo–O/S	Mo–O MoO_3_	S(-II) (1T′)	S(-II) (2H)	S_*n*_(-II)
1	10.5	6.9	5.7	6.7	22.1	13.2	34.8
3	17.3	6.5	4.3	3.6	29.3	12.3	26.8
5	20.1	6.3	3.4	1.6	36.6	10.7	21.3
10	21.8	6.3	3.4	1.7	38.7	10.1	17.9

a The relative contributions were calculated from integrated intensities after normalization to the corresponding Mo 3d and S 2p sensitivity factors.

**Table 4:** The 1T′/2H Ratio in As-deposited Amorphous MoS_2_ Thin Films Deposited at 1, 3, 5, and 10 Hz Derived from Both Mo 3d and S 2p XPS Spectra

	1 Hz	3 Hz	5 Hz	10 Hz
Mo 3d	1.5	2.7	3.2	3.5
S 2p	1.7	2.4	3.4	3.8

Comparison of the VB spectra for amorphous MoS_2_ deposited at 1, 3, 5, and 10 Hz reveals that the primary spectral features are essentially identical, as demonstrated in Figure 6B. The only significant distinction identified is the gradual increase in the density of states at the Fermi level observed in the MoS_2_ phase, which correlates directly with increasing deposition repetition rates.

In general, the specific local bonding and coordination of atoms are the critical factors determining the properties of amorphous chalcogenides.^44^ Here, we investigated the role of the 1T′/2H ratio in as-deposited amorphous thin MoS_2_ films on spectroscopic optical parameters. Figure 7 shows the resulting spectroscopic dependencies of refractive index, *n*, and extinction coefficient, *k*, for the corresponding MoS_2_ samples. The observed monotonic decrease of optical constants *n* and *k* for all thin MoS_2_ films with increasing wavelength and the absence of excitonic features strongly suggest that the MoS_2_ is in an amorphous or highly disordered noncrystalline phase, as opposed to the characteristic electronic and optical response of the crystalline counterpart. Notably, the spectral dependencies of both *n* and *k* significantly increase with an elevated concentration of 1T′ structural units, which incorporate homopolar Mo–Mo bonds. Furthermore, the substantially enhanced values of the spectral dependences could also be contributed by small Mo clusters, as revealed by XRD analysis in the films deposited at laser repetition rates of 5 and 10 Hz.

**Figure 7:**
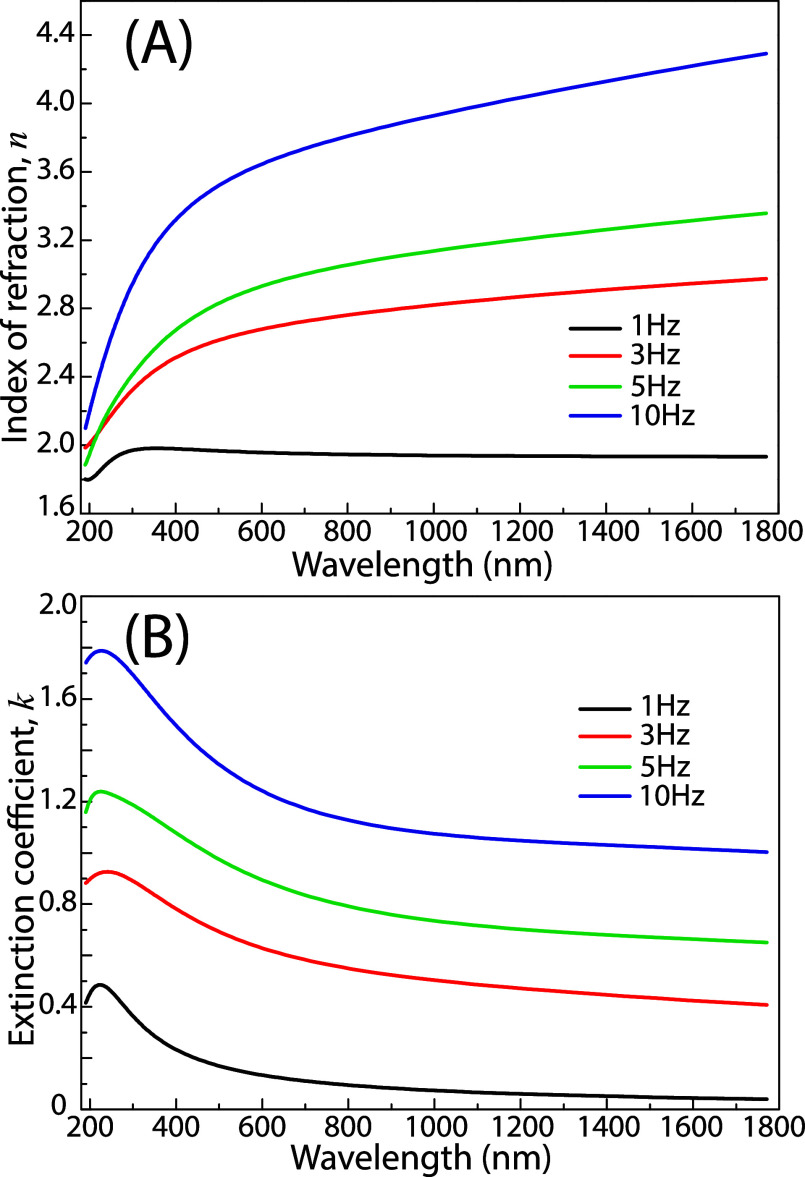
Spectroscopic optical parameters (A) refractive index, *n* and (B) extinction coefficient, *k* of as-deposited amorphous MoS_2_ thin films deposited at 1, 3, 5, and 10 Hz.

Spectroscopic ellipsometry also revealed the dependence of MoS_2_ film thickness and surface roughness on the laser repetition rate. The total film thickness transitioned from 99 nm at 1 Hz to a stabilized range of 36–44 nm at higher frequencies (3–10 Hz), suggesting a significant variation in the deposition rate per pulse. The determined thicknesses of the overlayer that correlate with surface roughness are 3, 8, 7, and 12 nm for MoS_2_ films deposited at 1, 3, 5, and 10 Hz, respectively. This morphological coarsening is in excellent agreement with the SEM micrographs in Figure 4, which depict a transition toward a more topographically irregular surface with pronounced roughness at higher frequencies.

Given the remarkably high *k* values exhibited by the thin MoS_2_ films deposited at high repetition frequencies, these materials are promising candidates for photothermal applications, such as photothermal cancer therapy^45,46^ and photothermal sterilization^47,48^ and desinfection.^49^ The amorphous MoS_2_ films and a fused silica (a control sample) were illuminated using a supercontinuum laser source to investigate their photothermal potential. The incident beam was spectrally restricted by a long-pass filter to the near-infrared (NIR) range of 1000–2200 nm with a total power of 340 mW. This chosen spectral region is highly relevant to biomedical applications as it encompasses both the NIR-II (1000–1350 nm) and NIR-III (1600–1870 nm) parts of the biological transparency window, where light penetration into tissue is maximized. The light-to-heat conversion (LHC) of the amorphous MoS_2_ thin films, measured as a function of deposition repetition rate, is presented in Figure 8. The clean fused silica substrate was utilized as a control sample. As expected, due to its transparency across the NIR spectrum used for irradiation, the substrate exhibited no measurable temperature increase. The amorphous MoS_2_ films deposited at the low repetition rate of 1 Hz (characterized by a low content of 1T′ units) exhibited only a slow and poor LHC response, reaching a saturation temperature difference, Δ*T*, of approximately 3 °C. Conversely, irradiation of the amorphous MoS_2_ films deposited at 3, 5, and 10 Hz resulted in a sharp, instantaneous increase in the recorded temperature immediately upon beam activation, followed by a gradual saturation to a thermal steady state. The maximum values of Δ*T* recorded were 13, 30, and 36 °C for films deposited at 3, 5, and 10 Hz, respectively. All Δ*T* values demonstrate excellent agreement with the observed trend in the corresponding *k* values, affirming the direct correlation between optical absorption and LHC efficiency. The recorded LHC dependences of the amorphous MoS_2_ film deposited by PLD (at 5 and 10 Hz) exhibit equally robust effectiveness to those previously reported on amorphous MoS_2_ deposited by magnetron sputtering with a similar thickness^50^ and MoS_2_ when stabilized in its 1T crystalline phase^51^ or via sulfur vacancy engineering of MoS_2_ in 2H modification.^48^ Due to both the simple tunability of deposition conditions for the amorphous phase and inherent thermodynamic stability relative to both the 1T and the sulfur-deficient 2H MoS_2_ structures, amorphous MoS_2_ emerges as a particularly promising candidate for photothermal applications.

**Figure 8:**
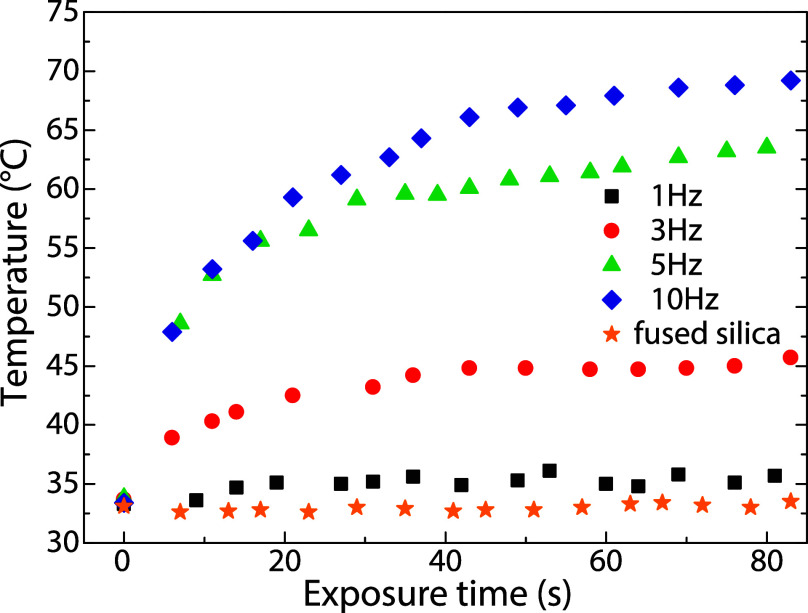
Photothermal conversion of the as-deposited amorphous MoS_2_ thin films deposited at 1, 3, 5, and 10 Hz.

## Conclusions

In summary, this study demonstrates how PLD conditions allow for precise and tunable control of the physical and chemical properties of amorphous thin MoS_*x*_ films. We established a critical link between the laser fluence (specifically from 1.3 to 20.4 J/cm^2^) and the MoS_4_ stoichiometry, allowing us to precisely tune the sulfur content from MoS_*x*_ to stoichiometric MoS_2_ at a fixed 1 Hz repetition rate.

Focusing on amorphous MoS_2_, we further revealed that while the stoichiometry remains constant, increasing the laser repetition rate (from 1 to 10 Hz) systematically drives a structural transformation. X-ray photoelectron spectroscopy confirmed a significant increase in the ratio of 1T′/2H structural units, concurrent with a reduction in polysulfide and undesirable molybdenum oxide impurities.

Importantly, these structural modifications in amorphous MoS_2_ translate directly into a marked increase in their refractive index and extinction coefficient with increasing laser frequency. This improvement was found to be systematically associated with higher photothermal conversion efficiency. Therefore, the PLD repetition rate represents a powerful tool for engineering the structure and optical properties of amorphous MoS_2_ and these highly tunable films are promising candidates for practical applications, particularly in photothermal cancer therapy and sterilization.
